# The role of motility and chemotaxis in the bacterial colonization of protected surfaces

**DOI:** 10.1038/srep19616

**Published:** 2016-01-21

**Authors:** Einat Tamar, Moriah Koler, Ady Vaknin

**Affiliations:** 1 The Racah Institute of Physics, The Hebrew University of Jerusalem, Safra Campus, Givat Ram, Jerusalem, Israel

## Abstract

Internal epithelial surfaces in humans are both oxygenated and physically protected by a few hundred microns thick hydrogel mucosal layer, conditions that might support bacterial aerotaxis. However, the potential role of aerotaxis in crossing such a thin hydrogel layer is not clear. Here, we used a new setup to study the potential role of motility and chemotaxis in the bacterial colonization of surfaces covered by a thin hydrogel layer and subjected to a vertical oxygen gradient. Using the bacterium *Escherichia coli*, we show that both non-motile and motile-but-non-chemotactic bacteria could barely reach the surface. However, an acquired mutation in the non-chemotactic bacteria that altered their inherent swimming behavior led to a critical enhancement of surface colonization. Most chemotactic strains accumulated within the bulk of the hydrogel layer, except for the MG1655 strain, which showed a unique tendency to accumulate directly at the oxygenated surface and thus exhibited distinctly enhanced colonization. Even after a long period of bacterial growth, non-motile bacteria could not colonize the hydrogel. Thus, switching motility, which can be spontaneously acquired or altered *in vivo*, is critical for the colonization of such protected surfaces, whereas aerotaxis capacity clearly expedites surface colonization, and can lead to diverse colonization patterns.

A hydrogel layer with a thickness of a few hundred micrometers covers most of the internal epithelial surfaces in humans^1^,^2^,^3^. This hydrogel consists of cross-linked mucin molecules and provides the first line of defense against bacterial invasion. Several cases were reported in which bacterial motility or chemotaxis could affect bacterial infection^4^,^5^,^6^,^7^,^8^. In particular, it was suggested that because the epithelial surfaces are oxygenated by the underlying bloodstream, an oxygen gradient is maintained across the mucin layer that can be exploited by bacterial cells for oxygen-driven taxis^9^,^10^,^11^ (aerotaxis), which might expedite the infection process. Clearly, the hydrogel layer can generally inhibit the otherwise free access of the bacterial cells to the surface. However, although the thickness of the layer is much larger than the size of individual bacteria, it is comparable to the size of a single bacterial colony, potentially making the role of bacterial growth more significant in this context. In addition, bacterial diffusion and active random walk are normally negligible over large distances; however, these factors could potentially play more significant role over the short distances and long time scales that are typical in this case. Finally, previous studies indicated that bacterial aerotaxis might actually inhibit the bacterial population from getting close to oxygen-rich surfaces^12^. Thus, given the intermediate thickness of this layer, the role of bacterial taxis capacity is not *a priori* clear in this context.

Many quantitative studies of bacterial chemotaxis have been conducted using a variety of behavioral assays^13^,^14^,^15^,^16^,^17^,^18^,^19^. However, none of these assays capture the basic constraints imposed by the mucosal layer: a wide-area hydrogel barrier, an intermediate thickness of the hydrogel, and an oxygen gradient that is maintained vertically across the layer. In addition, although the ability of bacterial cells to colonize both biotic and abiotic surfaces has been studied in various *in vitro* setups, most of these studies lack a physical barrier between the bacteria and the surface. Clearly, under such conditions, bacterial motility or chemotaxis capacity might play a fundamentally different role.

To study the role of bacterial motility and chemotaxis in the colonization of protected surfaces, we focused on the bacterium *Escherichia coli*, a common resident of the human gut^20^,^21^, whose chemotaxis system has been well studied at the molecular and behavioral levels. *E. coli* propels itself by rotation of long helical flagellar filaments that extend from its outer membrane and are powered by proton-driven motors^22^. If all of the flagella turn in a direction defined as counter-clockwise(CCW), they form a compact single bundle that propels the cell forward; however, if one or more flagella switch to the opposite direction (clockwise, CW), that flagellum leaves the bundle, and the cell switches its swimming direction. These two modes of swimming, referred to as ‘run’ and ‘tumble’, respectively, constitute an active random walk. Similar to all motile bacteria, *E. coli* is also equipped with a sensory system that detects external chemical changes along the bacterial swimming trajectory and guides the bacterium along chemical gradients, a behavior known as chemotaxis^23^. This sensory system consists of four types of MCP chemoreceptors with various sensing specificities and an additional MCP-like Aer receptor^24^. These chemoreceptors activate and regulate an associated histidine kinase (CheA), which in turn donates phosphoryl groups to a cytoplasmic response regulator CheY. A dedicated phosphatase CheZ removes the phosphoryl groups from CheY, thus allowing the intracellular level of phospho-CheY to rapidly follow changes in the external environment. The binding of phospho-CheY to the base of the flagellar motor biases its rotation towards CW rotation and thus promotes switching of the bacterial swimming direction. Sensory adaptation mediated by methylation or demethylation of the receptors by CheR and CheB, respectively, allows for time-resolved comparison of ligand concentrations and extends the dynamic range of the responses.

The capacity of *E. coli* cells to navigate along oxygen gradients (aerotaxis) relies on the Aer receptor, which detects the redox potential across the cytoplasmic membrane, as does the Tsr receptor^12^,^24^,^25^. However, whether *E. coli* seeks specific oxygen levels^26^,^27^ or generally seeks the highest level possible^28^ is still a topic of debate. The molecular mechanism of aerotaxis behavior is also not fully understood because the Aer receptor lacks methylation sites and thus is not subjected to the conventional adaptation mechanism^29^,^30^.

In this work, we report a new chemotaxis setup used to study the contribution of bacterial motility and chemotaxis to the ability of bacterial cells to colonize surfaces protected by a thin hydrogel layer subjected to a vertical oxygen gradient across the layer. Using this setup, we tested the capacity of different *E. coli* strains, including strains with specific motility or chemotaxis properties, to populate the hydrogel layer and colonize the surface.

## Results

### Bacterial surface colonization setup: the MG1655 strain as a test case

**Figure 1:**
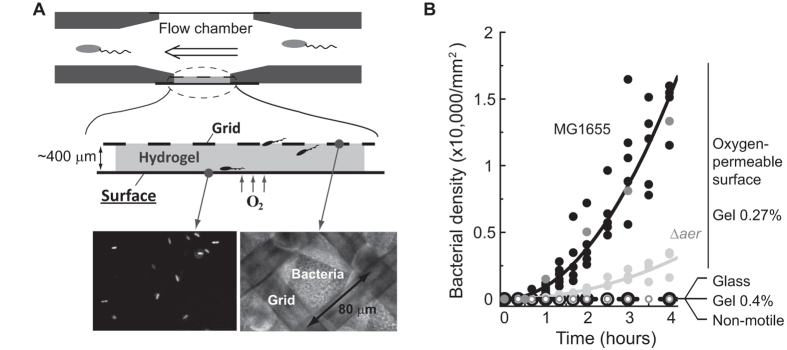
Surface colonization assay. (**A**) Schematic description of the setup. A thin hydrogel layer (0.27% Bacto-agar in motility buffer) approximately 400 μm thick was cast between a silicon-based oxygen-permeable surface (Paragon-HDS; Dk =58) and a nylon grid (80 × 80 μm^2^ square). This structure was subsequently placed in a titanium flow-chamber held at 30 °C, and the accumulation of bacterial cells was monitored using an inverted microscope. Also shown are sample images of the grid (right: transmission) and the surface (left: fluorescence). (**B**) Bacterial accumulation at the surface: MG1655 cells in a standard surface-down orientation of the chamber (filled black symbols); MG1655 cells in a surface-up orientation of the chamber (filled gray symbols); AVE3 (*aer*) cells (filled light gray symbols); non-motile UU1581 or AVE5 cells (black bars). Data from 3–5 independent experiments of each strain are presented. The accumulation of MG1655 cells near the surface was also tested with an oxygen-impermeable glass surface (open black symbols) or with a denser (0.4%) hydrogel (open gray symbols). Data from two independent experiments of each are presented.

The setup used to measure bacterial surface colonization in this work is shown in Fig. 1A (see Materials and Methods for a detailed description). A hydrogel layer (0.27% Bacto-agar) with a thickness of 350–450 μm was cast between an oxygen-permeable slide and a grid and subsequently placed in a titanium flow-chamber, which allowed for the continuous exchange of the medium above the gel. Given that the hydrogel layer is only a few hundred microns thick, diffusion of chemicals across the gel occurs on a time scale of several minutes and thus allows efficient exchange of the chemical environment in this layer. In addition, the hydrogel shields the cells in it from the flow, and thus cells are only carried in the flow above the grid but not underneath. The bacteria to be tested were suspended in a standard ‘motility’ buffer, which optimally supports bacterial motility and chemotaxis but does not support bacterial growth^13^. To induce a low level of oxygen in the cell suspension, after growing the cells to mid-exponential phase, we concentrate them in motility buffer to OD_600_=1. The bacterial suspension was then equilibrated in a closed tube and was drawn into the flow chamber from the bottom of the tube. Evidently, when such bacterial suspension was inserted into a capillary tube, cells at a distance larger than ~2 mm from the liquid/air interface did not swim, indicating that the oxygen level inside the cell suspension is very low^31^. Because oxygen enters through the oxygen-permeable surface and is consumed by the dense bacterial population in the flow chamber, an oxygen gradient forms across the hydrogel layer with a heightened oxygen level near the surface. However, the detailed oxygen profile is expected to be dynamically modified as the bacteria enter the hydrogel layer^15^,^31^. As shown below, the use of an oxygen permeable surface is evidently essential for observing surface colonization. The accumulation of cells at the surface and inside the gel layer was monitored using an inverted microscope with either transmitted light or fluorescence microscopy. Experiments were performed at 30 °C, a temperature commonly used in chemotaxis behavioral assays^13^.

As a test case, we first studied the behavior of the commonly used *E. coli* lab strain MG1655, which contains the insertion element *IS1* upstream of the motility master regulator *flhD*. The accumulation dynamics of these cells on the surface are shown in Fig. 1B. Each experiment began by initiating the flow of the bacterial suspension through the chamber, and the accumulation of cells at the surface was monitored over time as described in Materials and Methods. Bacterial cells could be detected at the surface 30–40 minutes after their introduction to the flow chamber and continued to accumulate over time (filled black symbols). In these experiments, as in most of the experiments reported in this work, the flow chamber was oriented with the oxygen-permeable surface pointing downward; however, similar bacterial accumulation was measured in experiments with the oxygen-permeable surface pointing upward (filled gray symbols).The bacterial cells could not reach the surface when the hydrogel density was increased from 0.27% to 0.4% (open gray symbols) or when non-motile bacteria were used (black bars). Next, we tested the importance of oxygen in the observed bacterial accumulation. First, we repeated the experiments with an oxygen-impermeable glass slide replacing the oxygen-permeable surface; in this case, no accumulation of cells could be detected (open black symbols). Second, we constructed a derivative strain of MG1655 that lacks the primary oxygen sensor Aer (AVE3); the accumulation of these mutant cells was significantly reduced (light-gray symbols). The AVE3 cells showed normal chemotactic behavior in standard semi-solid agar plates (0.27% Bacto-agar in TB), indicating that the chemotaxis capacity of the AVE3 strain is generally similar to that of the wild-type MG1655 strain when aspartate or serine gradients drive the colony expansion (Fig. S1). These observations indicate that oxygen indeed plays a key role in the process of surface colonization. Thus, the accumulation of bacterial cells on the surface was insensitive to the orientation of the flow chamber, blocked by higher gel densities, required bacterial motility, and critically depended on the presence of an oxygen-permeable surface and the Aer sensor.

### Surface colonization by various *E. coli* strains

**Figure 2:**
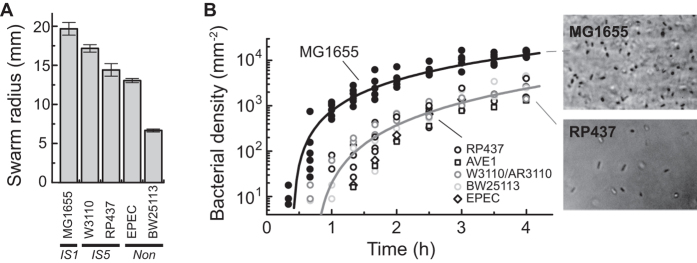
Surface accumulation of different E. coli lab strains. (**A**) Colony radius of the different strains used in this work in a semi-solid agar plate (0.27% Bacto-agar in TB) after 8 hours at 30 °C, averaged over 3 experiments with error bars representing SD. Strains are also labeled with the insertion element (*IS*) type found upstream of their *flhD* gene (Fig. S2). (**B**) Surface colonization dynamics of the different chemotactic strains. Between 2 and 5 repetitions are shown of the experiment with each strain on different days. Typical phase contrast images of the surface taken after 4 hours of accumulation with either the MG1655 or RP437 strain are also shown.

When tested in standard soft agar motility plates (see Materials and Methods), the colony expansion of commonly used *E. coli* lab strains varied widely (Fig. 2A). Because chemotaxis ability was previously shown to depend on insertion elements at the promoter region of the *flhD* operon^32^, we sequenced the *flhD* promoter regions of the specific strains used in this study and determined that the MG1655 strain carries an *IS1* element, the RP437 and W3110 strains carry the *IS5* element, and the BW25113 and EPEC strains lack an insertion element (Figs 2A and S2). When tested in the surface colonization setup, all of these strains showed similar surface colonization dynamics, with the exception of the MG1655 strain, which showed a distinct behavior (Fig. 2B); the MG1655 cells could be detected at the surface earlier and accumulated faster and to a higher level. Thus, under the conditions tested in this work, surface colonization is not sensitive to the variations among most strains except for the MG1655 strain, which had a clear advantage.

**Figure 3:**
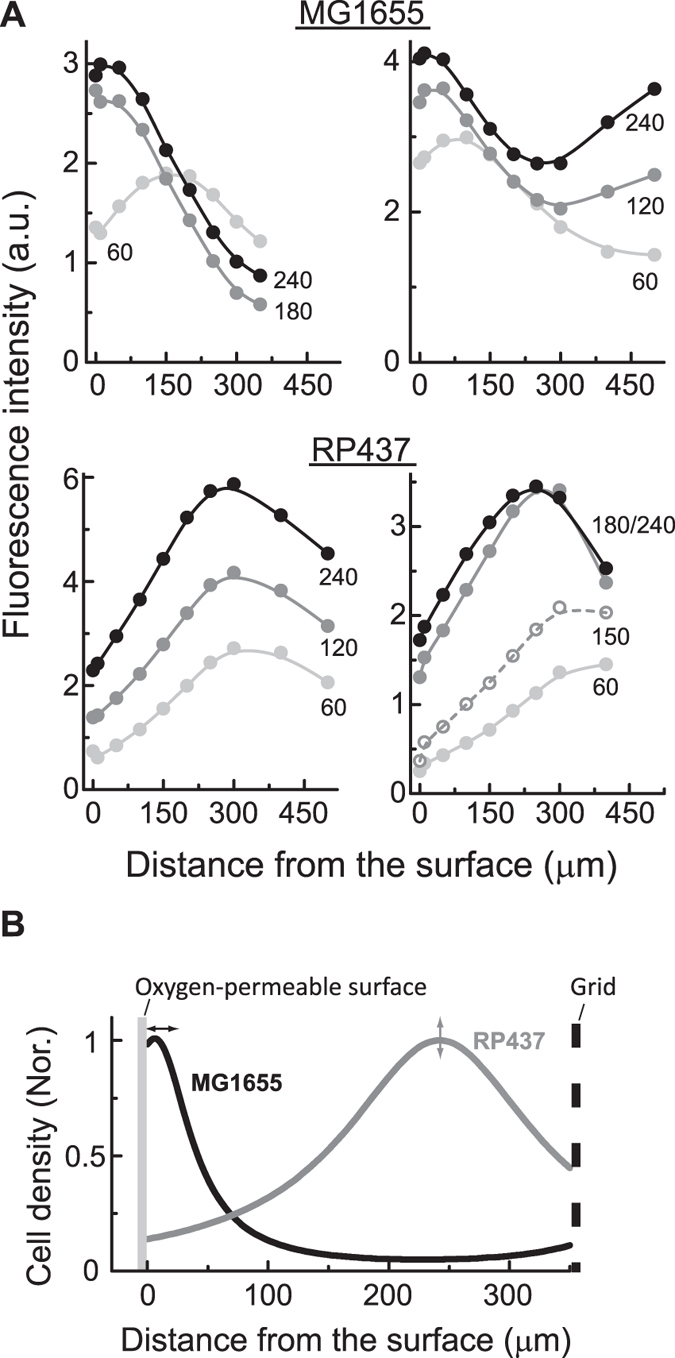
Bacterial distribution across the hydrogel layer. (**A**) Evolution of the fluorescence profile across the hydrogel layer (see *Materials and Methods*) for experiments with either the MG1655 or RP437 strain; two independent experiments with each strain are shown. For the MG1655 strain, profiles are shown for experiments conducted with the oxygen-permeable surface pointing downwards (right plot) or upwards (left plot). Each profile is labeled with the time elapsed since cells were applied to the flow chamber (in minutes). (**B**) Normalized cell distribution after 240 minutes for either MG1655 cells (black lines) or RP437 cells (gray lines) after 1D de-convolution analysis (see *Materials and Methods*).The arrows represent the margins of the fit variability, mostly in the peak position for the MG1655 strain and in the peak sharpness for the RP437.

To gain further insight into the unique behavior of the MG1655 strain, we compared the dynamics of its bacterial distribution across the hydrogel with that of other strains, primarily the RP437 strain. Bacterial distribution was evaluated by acquiring a series of fluorescence images at different distances from the surface and at different time points. The fluorescence intensity profiles were obtained by averaging the related images at each time point (see Materials and Methods). Figure 3A shows four sets of profiles (two of each strain) illustrating the progression of bacterial distribution within the hydrogel layer. Clearly, although cells of strain MG1655 had a strong tendency to accumulate directly at the oxygenated surface, cells of strain RP437 tended to accumulate within the bulk of the hydrogel layer. In the case of the MG1655 strain, examples are shown for experiments performed with the oxygen permeable surface pointing either downward (right plot) or upward (left plot). Notably, the additional accumulation of the MG1655 cells near the top of the hydrogel layer shown in the right-hand plot represents an accumulation of cells at the gel/flow interface; this accumulation was reduced when the flow-chamber was inverted. The profiles shown in Fig. 3A are intrinsically broadened by the optics. To extract more authentic distributions, we obtained intensity profiles from a defined thin bacterial layer and used this information to obtain more realistic distribution profiles using 1D-deconvolution (see Materials and Methods). The bacterial distributions obtained using this procedure are shown in Fig. 3B, further demonstrating the strong tendency of the MG1655 strain to accumulate near the surface (black lines) and the tendency of the RP437 strain to accumulate within the bulk of the hydrogel layer (gray lines). Thus, the rapid and enhanced surface colonization of the MG1655 strain shown in Fig. 2B clearly correlates with the strong tendency of these cells to accumulate directly at the oxygenated surface (see also Fig. S4).

Because the unique behavior of the MG1655 strain correlated with its unique insertion element (*IS1*) within the promoter region of the *flhD* operon^32^, we sought to test the possibility that the identity of the insertion element might be important. We replaced the *IS5* element in the RP437 chromosome with the *IS1* element from the MG1655 strain, resulting in strain AVE1 (Fig. S3). However, as shown in Fig. 2B (open squares) and in Fig. S4, the behavior of strain AVE1 was generally more similar to that of the parental RP437 strain and clearly different from that of the MG1655 strain. Thus, the *IS1* insertion element by itself is clearly not responsible for the unique behavior of the MG1655 strain. We also tested whether enhanced expression of the Aer sensor in the MG1655 cells leads to their unique behavior. This possibility was tested by supplementing the RP437 with plasmid pSB20 carrying the *aer* gene under an inducible promoter^24^; however, the enhanced expression of the Aer sensor did not significantly affect surface colonization (Fig. S5). Thus, the unique behavior of the MG1655 strain is not caused by its unique *IS1* insertion element or by enhanced expression of the Aer sensor.

### Analysis of motility and chemotaxis mutants

**Figure 4:**
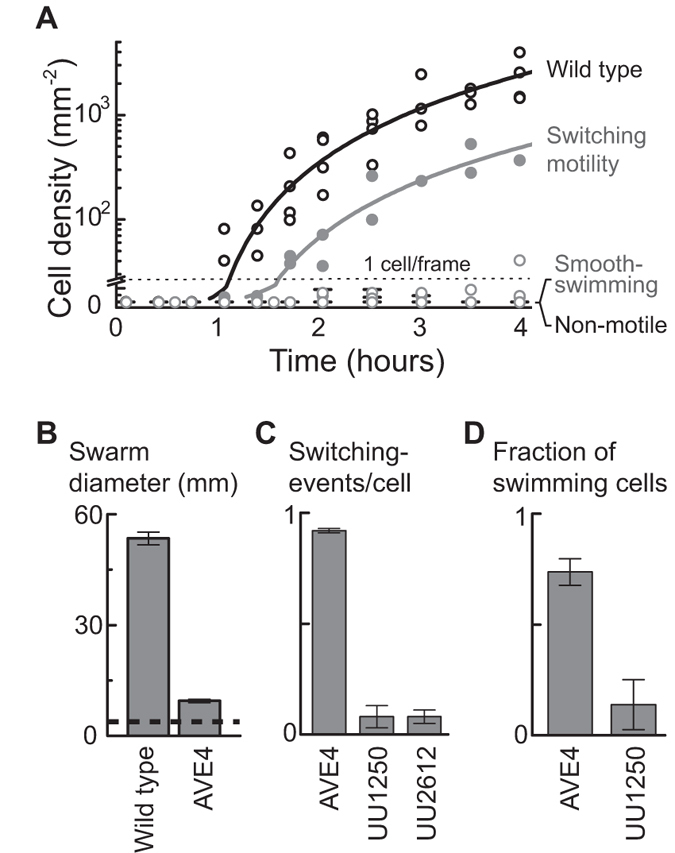
Motility and chemotaxis mutants. (**A**) Surface accumulation of strains with specific motility/taxis defects: the wild-type RP437 strain (open black symbols); the receptorless UU1250 and UU2612 strains as well as the AVE2 *cheY* strain (open gray symbols); the AVE4 strain, a derivative of the UU1250 strain that exhibits switching motility (filled gray symbols); and the non-motile UU1581 strain (black bars). The dotted line represents the threshold density below which less than one cell is expected in a frame. Data from 2–4 independent experiments with each strain are presented. Note the break in the abscissa. (**B**) Averaged colony diameter (over 3 experiments) of the AVE4 strain in standard semi-solid agar plates (0.27% Bacto-agar in TB) compared with that of its parental wild-type strain (RP437) measured from images taken after 11.5 hours at 30 °C. The dashed line represents the colony diameter of non-motile cells under the same conditions. (**C**) Number of switching events (tumble) per cell of free-swimming receptorless bacteria shown for different strains and extracted manually from short movies (50 successive frames with 0.1 s exposure time) recorded in motility medium. Approximately 50 cells (from 2–3 independent experiments) of each strain were analyzed. (**D**) Fraction of swimming bacterial cells counted in short movies (as in C) recorded from cells embedded in a bulk hydrogel. The data was averaged over 4 independent experiments in each approximately 100 cells were analyzed per strain; error bars represents SD.

As shown in Figs 1 and 2, despite the fact that the hydrogel is, in principle, accessible to bacterial cells, non-motile bacteria were unable to reach the surface, thus indicating that bacterial motility is essential for surface colonization under these conditions. To further identify the essential bacterial properties required to promote surface colonization, we analyzed derivatives of the RP437 and MG1655 strains that had specific motility and chemotaxis defects (Fig. 4A). The contribution of bacterial motility to surface colonization was tested using three motile but non-chemotactic strains; the UU1250 and UU2612 strains are derivatives of the RP437 strain that lack all known chemosensory receptors, and the AVE2 strain is a derivative of the MG1655 strain that lacks the response regulator CheY. As shown in Fig. 4A, these motile but non-chemotactic strains could barely reach the surface (open gray symbols), and, in fact, they exhibited a behavior comparable to that of the non-motile strains (black bars).

However, these non-chemotactic strains not only lack sensing capacity but are also expected to exhibit smooth-swimming behavior instead of the ‘run-and-tumble’ behavior (switching motility) of the wild-type cells. To test the role of bacterial chemotaxis in surface colonization separate from its effect on the intrinsic swimming traits of the bacteria, we used an additional strain that was obtained as follows. Following Wolfe *et al*.^33^, we inoculated cells of strain UU1250 (lacking all chemoreceptors) in a soft-agar plate and incubated the plates at 30 °C under strong selective pressure for cells with improved capacity to spread through the hydrogel. After approximately 48 hours of incubation during which the bacteria barely spread, clear out-grown bulges were observed. We isolated one of these mutants (AVE4) and confirmed that the spreading of this new strain in soft agar is indeed homogeneous (lacking bulges). Similar to the observation of Wolfe *et al*.^33^, cells of this AVE4 strain spread in soft agar only slightly better than non-motile cells and considerably less than wild-type chemotactic cells (Fig. 4B). Sequencing showed that this strain has a transposon insertion in the phosphatase *cheZ* gene. The lack of phosphatase activity, combined with the relatively long lifetime of phospho-CheY and a residual phosphorylation activity by CheA or acetyl phosphate can lead to a substantial level of phospho-CheY and thus to tumbling. Evidently, in contrast to the smooth-swimming behavior of the parental UU1250 strain, the AVE4 mutant bacteria exhibited run-and-tumble swimming behavior with frequent switching of their swimming direction (Fig. 4C). Moreover, quantification of the fraction of swimming cells within bacterial populations that were homogeneously mixed into the bulk of the hydrogel revealed that bacteria that were capable of switching their swimming direction tended to be more motile (Fig. 4D).When tested for surface colonization, the non-chemotactic AVE4 strain, which exhibit run-and-tumble swimming behavior (switching motility), colonized the surface far more efficiently than the parental non-chemotactic strain (Fig. 4A, filled gray symbols).

### Effect of bacterial growth on surface colonization: chemotactic vs. non-motile bacteria

In the surface-colonization experiments described thus far, the bacterial suspension was based on motility buffer, which was optimized for bacterial motility^13^, supports motility over extended periods, but does not support bacterial growth. This suspension was flown through the chamber for four hours while the bacterial surface accumulation was monitored. Thus, these experiments revealed the intrinsic capacity of bacteria to spread through the hydrogel layer and reach the underlying surface. However, when bacteria are allowed to grow, the physical expansion of the bacterial population by itself could potentially lead to spreading through the hydrogel. To test the effect of bacterial growth on surface colonization, we repeated the experiments mentioned above, but then, at the end of the four hours period, we continued the experiment by flowing fresh growth media (TB) through the chamber for additional 14 hours. At this second stage, suspended cells were washed out of the chamber and the trapped cells in the gel were allowed to grow.

**Figure 5:**
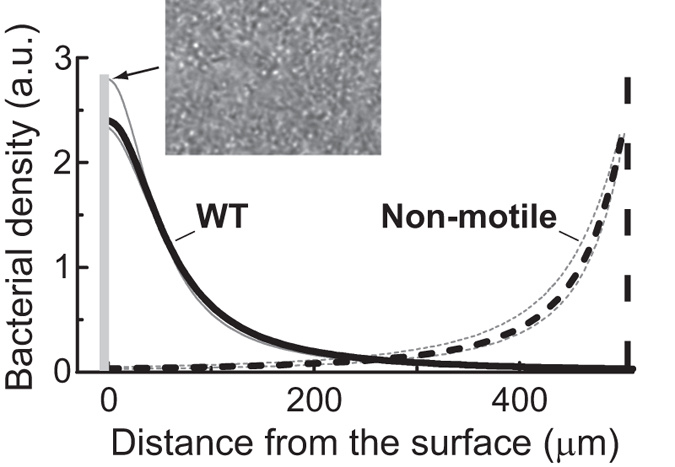
Bacterial distribution across the hydrogel layer after growth. Representative cell distributions (after 1D de-convolution) of the wild-type MG1655 cells (full line) or the non-motile UU1581 cells (dashed line) across the hydrogel layer following 4 hours of the standard surface-colonization experiment (in motility buffer) and an additional 14 hours of flowing fresh TB growth medium. The gray lines represent the margins of the fit variability.

As shown in Fig. 5, even after 14 hours of growth, non-motile bacterial cells could not be detected on the surface; moreover, although the outer gel/flow interface was heavily populated with bacteria, the bulk of the hydrogel layer was nearly depleted of bacteria. For comparison, when similar experiments were repeated with chemotactic MG1655 cells, the bacteria clearly populated the oxygen-permeable surface, forming a dense and flat bacterial layer. Interestingly, in the case of MG1655 cells as well, the bulk of the hydrogel layer was nearly depleted of bacteria (Fig. 5). Thus, despite its potential accessibility to the bacterial cells, the thin hydrogel layer was efficient at keeping the non-motile bacteria away from the surface even after an extensive growth period, thus leaving the surface nearly sterile.

## Discussion

Several factors could potentially contribute to the ability of bacterial cells to cross a thin protective hydrogel layer and colonize the underlying surface: passive diffusion, growth-driven expansion, random motility, and chemotaxis. Although random motility and growth-driven expansion are both negligible over large distances, these processes might be potentially relevant over the short-length scale typical of the hydrogel mucosal layer and the time scale typical for the *in vivo* regeneration time of this layer (few hours). For example, the effective diffusion coefficient of free swimming *E. coli* cells conducting a random walk was estimated to be on the order of 4·10^−6 ^cm^2^/sec and thus can effectively expand over ~300 μm in only a few minutes but would require ~1,000-fold longer time to expand over one centimeter^22^. In addition, the size of the colony formed by non-motile bacteria in soft agar (in TB at 30 °C) can reach a size of several hundred microns. Thus, the contribution of the different bacterial properties to surface colonization under these conditions is not *a priori* clear. To study the potential contribution of these bacterial properties to surface colonization under these conditions, we developed a setup for studying the ability of bacteria to colonize surfaces protected by a thin hydrogel layer subjected to a vertical oxygen gradient.

### Role of bacterial growth

In principle, the pressure formed within a confined bacterial colony could drive the expansion of cells through the soft hydrogel, which is evidently accessible to these bacteria. Under the conditions described in these experiments a single colony grown for 14 hours within a TB-based soft agar can reach a diameter of 150–200 microns. However, despite the continuous supply of fresh growth medium by the flow in the channel assay and the large number of bacteria that colonized the upper gel-flow interface, non-motile bacteria were essentially undetected within the bulk of the hydrogel layer or at the underlying surface even after 14 hours (Figs 1 and 5). Note that even in the *in vivo* system, regeneration of the mucin hydrogel provides a natural time limit of several hours^34^. Thus, under the conditions tested in this work, despite its general potential, growth-driven expansion is insufficient to promote bacterial colonization or substantial colonization of the thin hydrogel layer. This behavior might be related to the fact that in contrast to the bulk hydrogel, the hydrogel/flow interface is intrinsically asymmetric and allows shedding of bacterial cells into the flow; thus, it is not conducive to the expansion of the bacterial into the bulk of the hydrogel.

### Role of switching motility

Motile bacteria that lack chemotactic capacity were deficient at surface colonization (Fig. 4), indicating that motility by itself is not effective in promoting bacterial spreading even through a thin hydrogel layer. However, in addition to affecting taxis capacity, defects in the chemosensory system also modify the intrinsic swimming behavior of the bacteria, exhibiting smooth-swimming behavior instead of the switching motility (run-and-tumble) exhibited by the wild-type cells. This is generally expected because the default state of the flagella motor is CCW rotation in the absence of a functional sensory system that phosphorylates CheY. As noted by Wolfe *et al*.^33^ (see also Fig. 4B), this ability of the bacterial cells to actively switch their swimming direction promotes somewhat more efficient spreading of the colony in semi-solid agar plates. However, because of the short distance across the hydrogel layer, cells that exhibited switching motility (strain AVE4), even in the absence of sensing capacity, showed a dramatic improvement in their capacity to colonize the surface over that of the smooth-swimming bacteria (Fig. 4). This observation suggests that the behavior of non-chemotactic strains in an *in vivo* context should be interpreted cautiously, particularly because inherent changes in swimming traits can be acquired by spontaneous mutations. Moreover, it might be expected that environmental factors within the hydrogel layer that could affect the tumbling rate of non-chemotactic bacteria can potentially affect their capacity to cross the protective hydrogel layer. For example, acetate (a common metabolic product in the gut) or factors that modulate the cyclic-di-GMP level in the cell can affect the rotation bias of the flagellar motor and thus might affect surface colonization^35^,^36^.

The effect of switching motility on the ability of non-chemotactic bacteria to spread through the hydrogel is correlated with their ability to swim vigorously within the hydrogel (Fig. 4D), despite the fact that their intrinsic swimming speed in liquid was not altered. As suggested by Wolfe *et al*.^33^, the hydrogel is a non-homogeneous porous environment in which smooth-swimming bacteria might be more likely to become trapped. However, additional reasons for the advantage of switching motility are also possible. For example, a direct effect of oxygen on swimming speeds might have stronger effect on cells with an appropriate switching rate, induced by mutations. In addition, it was recently observed that the flagellar motors can undergo remodeling in response to changes in either the phospho-CheY level or a load that can affect the output torque^37^,^38^. It is possible that increased tumbling by itself affects the output torque of the flagellar motor and thus allows these bacteria to swim more efficiently within the hydrogel^39^.

### Role of chemotactic capacity

The role of chemotaxis in surface colonization is clearly demonstrated in the following examples. First, the accumulation of the RP437 strain is more rapid and enhanced compared with that of its non-chemotactic derivative strains, including strain AVE4 that exhibits switching motility (Fig. 4). Second, the accumulation of the MG1655 strain is clearly more rapid and enhanced compared with that of its *aer* derivative (Fig. 1), which lacks the primary oxygen sensor but maintains normal chemotaxis capacity in soft-agar plates (Fig. S1). This substantial effect of the Aer sensor further indicates that aerotaxis is playing a significant role in the observed surface accumulation. Note that the Tsr sensor can contribute to the residual bacterial accumulation in the absence of Aer^12^. The role of aerotaxis is further supported by the fact that replacing the oxygen-permeable surface with oxygen-impermeable slide abolished the bacterial accumulation (Fig. 1). The permeability of the surface to oxygen on the one hand, and the low oxygen level induced by the bacterial suspension on the other hand, are expected to lead to an oxygen gradient across the gel layer, in a similar way as in ref. 12. Evidently, when the bacterial suspension was tested in a capillary tube, cells were not swimming at a distance larger than ~2 mm from the liquid/air interface, indicating that the oxygen level there is low^31^. However, near the oxygen-permeable surface, bacterial cells were vigorously swimming, indicating that the oxygen level near the surface is clearly elevated. A simple estimate of the expected change in the oxygen concentration across the gel layer, suggests that this change can be larger than 30% (see Supplementary). Given that the half-maximal response of the cells to oxygen can be as low as 0.7 μM^40^ and that the bacterial typical ‘run’ (swimming) length is 10 μm, the oxygen gradient that can potentially trigger an aerotaxis response can be less than 0.07 μM/μm, corresponding to a difference of approximately 30 μM oxygen across the 400 μm of the gel layer. This difference is approximately 10% of the oxygen concentration at the air/liquid interface. Thus, aerotaxis appears to be indeed feasible under the conditions tested here. Clearly, additional factors might also play a role in the observed surface colonization, particularly in the experiments in which bacterial growth is involved (Fig. 5). In this case, the nutrients in the growth medium or even chemicals produced by the bacteria^41^ might also affect their distribution, which becomes sharper at the end of the growth period.

Comparing the MG1655 strain with other chemotactic strains (Fig. 2) revealed a unique behavior of the MG1655 strain in that it rapidly approached the oxygenated surface and accumulated directly at the surface, whereas the RP437 cells, similar to other strains, tended to accumulate within the bulk of the hydrogel layer (Fig. 3). This tendency of the MG1655 strain also led to a clear enhancement in its ability to approach the surface. The different distributions of the different strains across the hydrogel persisted over time and thus appear to reflect a qualitatively different preference of the two strains rather than a dynamic effect. Thus, chemotactic capacity not only enhances surface colonization but also offers a means for diversifying and controlling the spatial organization of the bacteria within such environments.

What makes the behavior of the MG1655 strain unique? A well characterized motility-related difference between the MG1655 strain and all of the other strains tested in this work is the unique presence of the *IS1* insertion elements upstream of the *flhDC* operon, which encodes the motility master regulators (Figs 2A and S2). It was previously shown that various insertions in this location can lead to different chemotactic behaviors^32^. However, the behavior of the AVE1 strain, a derivative of the RP437 strain that carries the *IS1* insertion element instead of its original *IS5* element upstream to the *flhD* gene, was still essentially similar to that of its parental RP437 strain (Figs 2 and S4). Thus, the *flhDC* regulatory region does not cause the unique behavior of the MG1655 strain. Another candidate is the Aer sensor, which significantly contributes to surface colonization under the conditions tested in this work (Fig. 1). However, elevation of the expression of this sensor in the RP437 cells still could not promote a behavior similar to that of the MG1655 strain (Fig. S5). In an attempt to identify additional candidates for the different behaviors of the specific strains used in this work, we conducted a whole-genome search for mutations in the RP437 strain relative to that of the MG1655 strain (Table S2). We subsequently compared these mutations with the documented differences between the W3110 and MG1655 strains^42^ by searching for mutations in similar loci in both RP437 and W3110 but not in MG1655. As candidates, this search identified the *crp* gene (the regulator of catabolic repression) and the *rpoS* gene (the dominant sigma factor in stationary phase), both of which were mutated in the RP437 and W3110 strains compared with the MG1655 strain (see Supplementary Table 2). Both genes can indeed affect bacterial motility^43^,^44^,^45^. However, because this analysis does not include genomic insertions, additional candidates are also possible, and the mechanistic explanation for the distinct behavior of the MG1655 strain awaits further study.

### Hydrogel and the bacterial dilemma

Bacterial motility is generally not conducive to the expression of genes that promote bacterial adherence^46^. Such opposing regulations, together with the observation that motility can play a critical role in surface colonization (Fig. 5), suggest that the protective hydrogel layer presents an intrinsic dilemma to the bacteria related to their capacity to access the epithelial surface and their capacity to adhere to epithelial cells. Thus, when an heterogeneous bacterial population is faced with the challenge of surface colonization, cells that are prone towards the sessile state and could potentially initiate efficient contact with the epithelial surface are expected to have lower probability of actually approaching the surface, and vice versa; cells that are highly motile can efficiently approach the epithelial surface, but their capacity to adhere to the surface is expected to be lower. Such intrinsic conflict might require physiological adaptation of bacteria during the colonization process, which might rely on specific cues within the mucosal layer. The setup presented in this work could be used to study such interactions between bacteria and epithelial surfaces while maintaining the requirement that the bacteria must first cross the hydrogel layer prior to contact with the surface.

## Materials and Methods

### Bacterial surface colonization setup and experimental procedure

The setup used to measure bacterial surface colonization in this work is shown in Fig. 1A. A hydrogel layer with a thickness of 350–450 μm was cast between an oxygen-permeable slide and a nylon grid with an 80 μm mesh size (80 μm mesh size, SPI supplies). The hydrogel layer used in the experiments reported in this work was based on the 0.27% Bacto-agar gel commonly used in chemotaxis and motility studies. The oxygen-permeable surface was made from a flat, rigid, transparent, and biocompatible silicon-based surface (Paragon-HDS, 58 Dk; Soflex) that is routinely used in commercial contact lenses. After solidification, this structure was subsequently placed in a titanium flow-chamber (volume: 1 ml) that allowed continuous exchange of the medium in the chamber. The flow chamber was mounted in an inverted microscope (Nikon Ti) equipped with a homemade temperature-controlled stage insert, and the temperature was set to 30 °C.

The bacteria to be tested were grown overnight at 30 °C in 2 ml of TB (10 g/l Bacto-Tryptone, 5 g/l NaCl) supplemented with 100 μg/ml ampicillin. Overnight cultures were then diluted 50-fold in 100 ml of TB supplemented with 100 μg/ml ampicillin and 100 μM IPTG, and allowed to grow aerobically at 33.5 °C. When cultures reached an OD_600nm_ of 0.45 cells were washed twice in 10 ml of motility buffer (0.01 M KPO_4_, 0.1 mM EDTA, 0.067 M NaCl, 1 μM methionine, and 0.01 M lactic acid, in 1 L DDW, pH 7.0) and gently re-suspended into 6 ml of motility buffer via slow rocking. Cells were washed without re-suspension in between washings to minimize potential motility damage; however, any residual traces were extensively diluted by this washing procedure. Finally, motility buffer was added to form a bacterial suspension with OD_600_~1. The final cell suspension was slowly (0.1 ml/min) flowed through the chamber. The accumulation of cells at the surface and inside the gel layer was monitored using a 40X air objective via either transmitted light or fluorescence microscopy. The microscope was equipped with a temperature-controlled stage set to 30 °C, a temperature commonly used in chemotaxis behavioral assays^13^. Bacterial surface accumulation was routinely followed for approximately four hours. During this time, the bacterial colonization of the underlying silicon surfaces and their distribution profiles were analyzed as described below. For a few strains, at the end of this taxis experiment, clean motility buffer was flowed through the chamber for 30 min to wash the suspended cells, and fresh TB supplemented with 100 μg/ml ampicillin and 100 μM IPTG was subsequently flowed through the flow chamber for an additional 10–14 hours (0.05 ml/min), after which the surface colonization and profile were analyzed.

### Strains, plasmids, and growth conditions

Five commonly used *Escherichia coli* lab strains were tested in this work (Table S1): MG1655 (*IS1*^+^, V. Sourjik, Heidelberg University), RP437 (J. S. Parkinson, University of Utah), W3110 (R. Hengge, Free Berlin University), BW25113 (Keio collection), and EPEC (E2348/69, I. Rosenshine, Hebrew University). In addition, we used mutants with specific motility or chemotaxis defects (see also Table S1): the UU1581^47^ and AVE5 (*fliC*) are non-motile, the UU1250^48^ and UU2612^49^ receptorless strains and the AVE2 (*cheY*) strain are non-chemotactic, AVE3 (*aer*) is deleted for the primary oxygen sensor, and AVE1 strain is a derivative of the RP437 strain in which the original *IS5* element upstream of the *flhD* gene was replaced by the corresponding *IS1* element from strain MG1655 (see Fig. S3). In addition, strain AVE4 is a derivative of the UU1250 strain that was selected (this study) for increased spreading on a semi-solid agar plate. This strain was characterized by sequencing and was found to carry a Tn5 transposon in the *cheZ* gene. Strains UU1250, UU1581 and UU2612 are derivatives of the RP437 strain (Parkinson J S, University of Utah). Strain AVE5 (*fliC*) is a derivative of the JW1908 strain (Keio collection) with the kanamycin resistance cassette removed. The AVE2 (*cheY*) is a derivative of the MG1655 strain constructed by P1 transduction from JW1871 (Keio collection) and subsequent removal of the kanamycin resistance cassette. The AVE3 (*aer*) is a derivative of the MG1655 strain and was constructed by P1 transduction from JW3043 (Keio collection) and subsequent removal of the kanamycin resistance cassette. All strains were transformed with plasmid pAV41 carrying free *myfp* (*eyfp*^A206K^) and were induced with 100 μM IPTG.

### Surface colonization measurements

To evaluate the dynamics of bacterial accumulation on the surface, fluorescence images (5 s exposure time) were taken at 3 to 6 different random locations on the surface every 20–30 minutes, and the average number of cells bound to the surface was calculated for each time point. The bacterial distribution across the hydrogel layer was evaluated from a similar set of fluorescence images (1 s exposure time) taken at increasing heights above the surface (with 50 μm intervals). Such a set of measurements was acquired once every 30 minutes. The integrated fluorescence intensity was calculated for each image and plotted as a function of the distance from surface (z) (Fig. S6, blue symbols). To correct for the optical resolution, we constructed a defined thin layer (~50 μm) of bacterial cells in a hydrogel and measured its fluorescence profile in the same manner as described above. The obtained intensity profile could be fit by a Lorentzian *G*(*z* − *z’*) (inset of Fig. S6). Thus, for each bacterial distribution *F*(*z*), the expected measured intensity profile *I*(*z*) is a weighted sum of the fluorescence of each cell layers, or


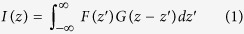


Using the measured *I*(*z*) and *G*(*z* − *z’*) and equation *Q1*, we could extract *F*(*z’*) by looking for the *F*(*z’*) that provides the *I*(*z*) that best fits the data after integration. In all cases, the obtained cell distribution was qualitatively verified by direct imaging.

### Semi-solid agar plates

From an overnight culture, 1 μl of cell suspension was inoculated in a semi-solid Bacto-agar hydrogel (0.27% Bacto Agar in TB, supplemented with 50 μg/ml ampicillin) poured in a 140 mm culture plate. The expansion diameter of the colony in the hydrogel was measured after 12 hours of incubation at 30 °C.

### Analysis of bacterial swimming behavior

Cells of the UU1250, UU2612 and AVE4 strains that were grown overnight were diluted 1:100 in TB and allowed to grow at 33.5 °C up to OD_600nm_ ≈ 0.45. The cells were washed and gently re-suspended in motility buffer to an OD_600nm_ of 0.3. For measurement of the bacterial intrinsic switching rate, the bacterial suspension was placed in a titanium chamber with a glass bottom and phase-contrast movies (50 frames, 0.1s exposure time each) were collected to track the bacterial swimming inside the chamber (at 30 °C). The average number of tumble events per cell was counted in each movie. A tumble event was counted if the cell abruptly switched its swimming direction. To measure the fraction of swimming bacteria in the hydrogel, the bacterial cells were mixed in the hydrogel (0.27% Bacto-agar in motility buffer) prior to its solidification, placed in the same titanium chamber and allowed to solidify. Similar movies were collected, and the relative number of swimming cells was counted.

### Sequence analysis

Starting from an overnight culture, cells of strains MG1655 and RP437 were diluted 1:100 and re-grown to an OD_600_ of 0.4; afterward, genomic DNA was extracted using a ‘DNeasy Blood and Tissue’ kit (QIAGEN). The DNA samples were sequenced at the Laboratory for Whole Genome Sequencing (Hadassah Medical School, The Hebrew University) using a Nextera-XT kit to prepare the DNA libraries and a Miseq sequencer (250 × 2 V2 kit), and samples were subsequently analyzed in the Galaxy environment. The sequencing data from each strain were aligned using the BWA mapping tool with the genome of *E. coli* MG1655 strain (K-12, version NC_000913.3) as the reference genome. The SNPs were identified using the SAM tools with thresholds set to coverage ≥4, and frequency >70%. Out of all identified SNPs, 67 amino acid replacements and 3 intergenic mutations were identified (Table S2). The identified mutations were compared with the known mutations in strain W3110^42^ to identify genes mutated in both RP437 and W3110 compared with MG1655.

## Additional Information

**How to cite this article**: Tamar, E. *et al*. The role of motility and chemotaxis in the bacterial colonization of protected surfaces. *Sci. Rep.* **6**, 19616; doi: 10.1038/srep19616 (2016).

## Supplementary Material

Supplementary Information
