# Supplementary material for: The role of motility and chemotaxis in the bacterial colonization of protected surfaces
Source: Sci Rep. 2016 Jan 21;6:19616. doi: 10.1038/srep19616 (PMC4726332; doi:10.1038/srep19616)
Supplement: Supplementary Information [file srep19616-s1.pdf]

The role of motility and chemotaxis in the bacterial colonization of protected surfaces

Einat Tamar, Moriah Koler, and Ady Vaknin

The Racah Institute of Physics, The Hebrew University of Jerusalem, Safra Campus, Givat Ram, Jerusalem, Israel

Supplementary Materials

Supplementary - Figure S1

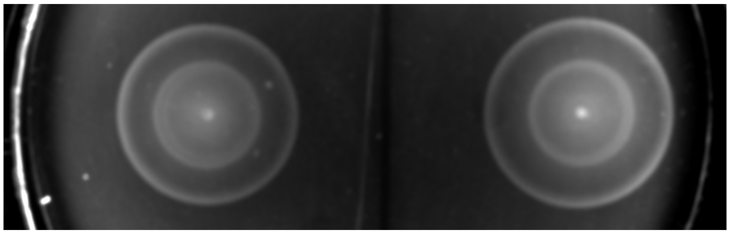

Fig S1. *Swarming assay of strain AVE3 (aer) compared to the wild type***.** Cells of strain AVE3 (right) or wild type MG1655 (left) were inoculated from an overnight culture into a common soft agar plate (see Materials and Methods). The image was taken after 7-8 hours of incubation at 30°.

Supplementary - Figure S2

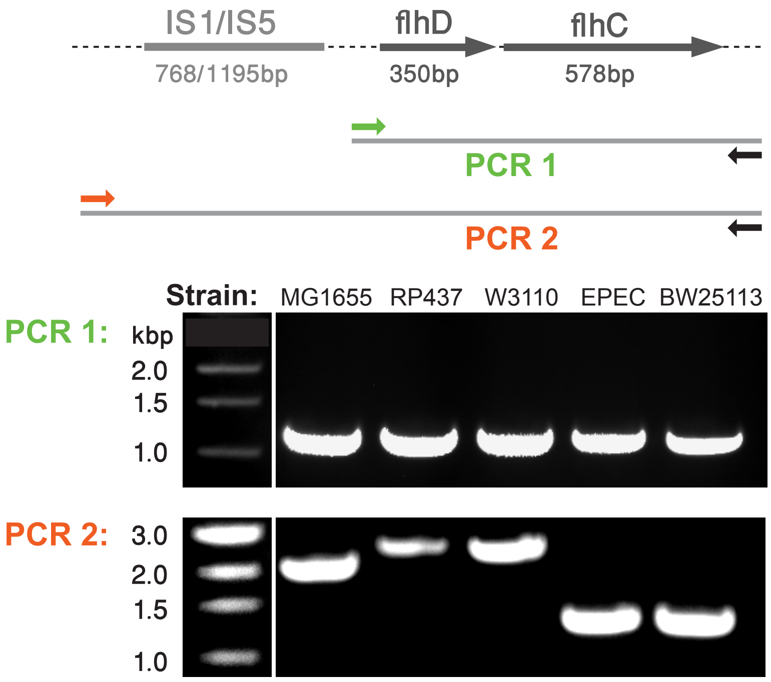

Fig S2. *Insertion elements characterization***.** We used two PCR reactions in order to identify the type of insertion element (IS1, IS5 or no element) located in the regulatory region of the *flhD* operon in the common lab strains: MG1655, RP437, W3110, EPEC(2348/69) and BW25113. The primers used are: CGTTACCGCTGCTGGAATGTTGCGCCTCAC (black), GCCCCCCTCCGTTGTATGTGCGTGTAG (green), and GACTGTGCGCAACATCCCATTTCGATTATTCCTG (red).

Supplementary - Figure S3

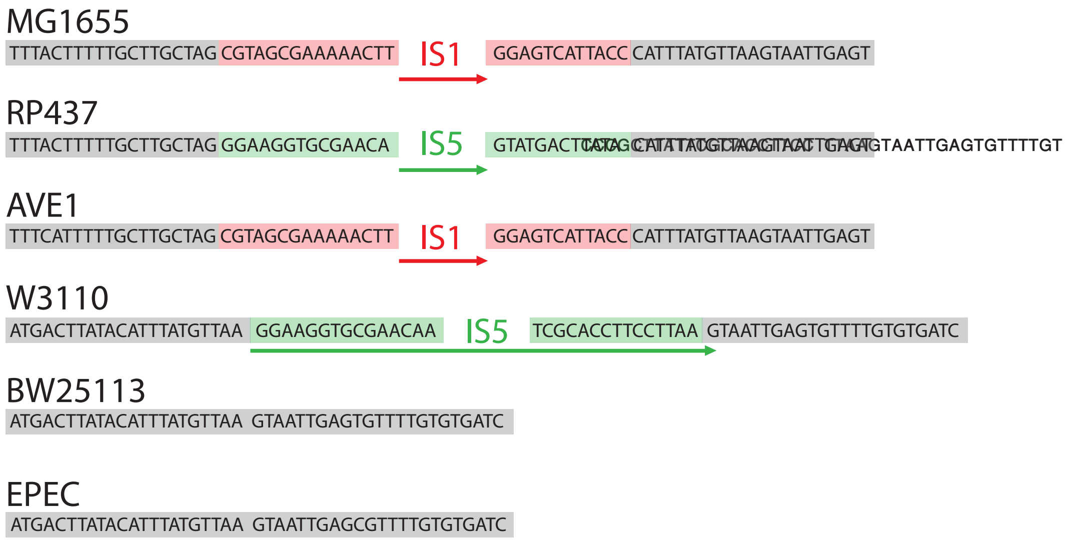

Fig. S3. *Sequenced insertion upstream of the flhD muster regulator in the various E. coli strains used in this study*. The outer boundaries of the sequenced insertion elements in strains MG1655, RP437, and *AVE1* are shown. Strain AVE1 was derived by replacing the region upstream of *flhD* with the corresponding region from the MG1655 strain. The region of the IS5 insertion in strain W3110 is compared with the corresponding region of strains BW25113 and EPEC, which lack the insertion (arrow).

Supplementary - Figure S4

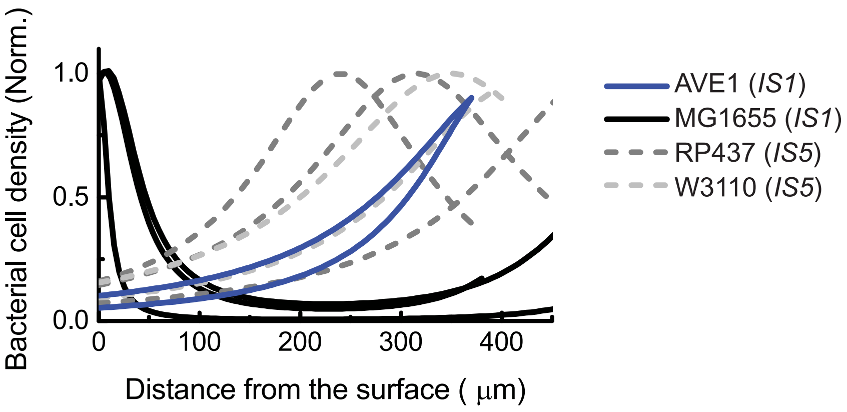

Fig S4. *The bacterial distribution across the hydrogel layer of strain AVE1 (RP437-IS1).* The normalized cell distribution 240 minutes after inoculation of strain *AVE1* carrying *IS1* upstream to *flhD* gene (solid blue lines) compared with the corresponding distributions obtained from the parental strain RP437 (*IS5*, gray dashed lines), strain MG1655 (*IS1*, solid black lines), and W3110 (*IS5*, light gray dashed lines). Distributions were derived as described in Materials and Methods and Fig. S6.

Supplementary - Figure S5

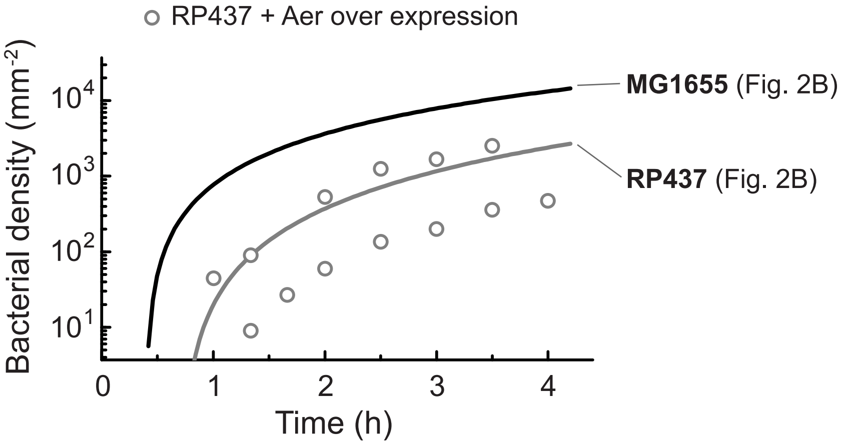

Fig S5. *Surface accumulation of RP437 cells over expressing the Aer receptor*. The surface colonization of RP437 cells transformed with a plasmid carrying the *aer* receptor (pSB20) under the control of the *lac* promoter, induced with 100µM IPTG. Lines are the fit to the corresponding data of strains MG1655 and RP437 (taken from Fig. 2).

Supplementary - Figure S6

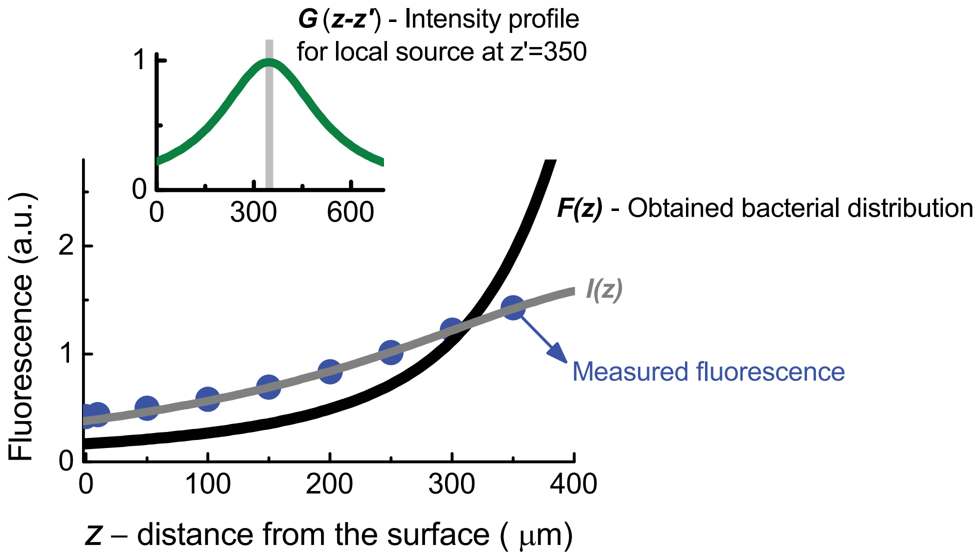

Fig S6. *1D-deconvolution procedure. Blue symbols* – The fluorescence profile *I(z)* obtained by integrating the fluorescence intensity for the corresponding images at different heights from the surface. *Black line* – The cell distribution *F(z)* obtained from *I(z)* by using Eq. 1 (see Materials and Methods). *Inset* – The fluorescence profile $G(z\text{-}z')$ obtained from a defined bacterial layer (~50μm thick) at position z'.

Table S1 (list of strains)

Strain	Description		Source
MG1655	Wild type	Chemotactic	Victor Sourjik
RP437	Wild type	Chemotactic	Sandy Parkinson
W3110	Wild type	Chemotactic	Regine Hengge
BW25113	Wild type	Chemotactic	Keio collection
EPEC	E2348/69, O127:H6	Chemotactic	Ilan Rosenshine
UU1581	*Δ(flhD-flhB), tsr, trg*	Non-motile	Sandy Parkinson[1]
UU1250	*tsr, Δ(tar-tap), trg, aer*	Non -sensing	Sandy Parkinson[2]
UU2612	*tsr, Δ(tar-tap), trg, aer*	Non -sensing	Sandy Parkinson[3]
JW1908	*fliC::kan*<br>(in-frame, single-gene knockout mutant of BW25113)	Non-motile	Keio collection[4]
JW1871	*cheY::kan* /BW25113	Non -sensing	Keio collection[4]
JW3043	*aer::kan* /BW25113	Chemotactic	Keio collection[4]
AVE1	A derivative of the RP437 strain carrying the IS1 insertions element upstream of the *flhD* gene.[5]	Chemotactic	This study
AVE2	Δ*cheY* /MG1655<br>In-frame deletion made by P1 transduction from JW1871 (Keio collection[4]) and subsequent removing of the kanamycin resistance cassette.	Non-chemotactic	This study
AVE3	Δ*aer* /MG1655<br>In-frame deletion made by P1 transduction from JW3043 (Keio collection[4]) and subsequent removing of the kanamycin resistance cassette.	Chemotactic (*Δaer)*	This study
AVE4	UU1250 strain carrying a partial TN5 insertion in *cheZ.* A spontaneous mutation selected on a swarm plat.	Non- sensing / switching motility	This study
AVE5	*ΔfliC* / BW25113<br>Derived from strain JW1908[4] , by removing of the kanamycin resistance cassette.	Non motile	This study

1 Studdert, C. A. & Parkinson, J. S. Crosslinking snapshots of bacterial chemoreceptor squads. *Proc. Natl. Acad. Sci. USA* **101**, 2117-2122, doi:10.1073/pnas.0308622100 (2004).
2 Ames, P., Studdert, C. A., Reiser, R. H. & Parkinson, J. S. Collaborative signaling by mixed chemoreceptor teams in *Escherichia coli*. *Proc. Natl. Acad. Sci. USA* **99**, 7060-7065 (2002).
3 Zhou, Q., Ames, P. & Parkinson, J. S. Biphasic control logic of HAMP domain signalling in the *Escherichia coli* serine chemoreceptor. *Mol. Microbiol.* **80**, 596-611, doi:10.1111/j.1365-2958.2011.07577.x (2011).
4 Baba, T. *et al.* Construction of Escherichia coli K-12 in-frame, single-gene knockout mutants: the Keio collection. *Mol. Syst. Biol.* **2**, doi:http://www.nature.com/msb/journal/v2/n1/suppinfo/msb4100050_S1.html (2006).
5 Barker, C. S., Prüß, B. M. & Matsumura, P. Increased motility of *Escherichia coli* by insertion sequence element integration into the regulatory region of the flhD operon. *J. Bacteriol.* **186**, 7529-7537, doi:10.1128/jb.186.22.7529-7537.2004 (2004).

Table S2 (RP437 mutations relative to MG1655)

Position in MG1655 genome	MG1655 base	RP437 base	Coverage	Percentage	Gene name	Amino acid replacement
3730560	C	T	40	100	*xylA*	W->stop
3647428	C	T	40	100	*gor*	A->V
3581222	A	G	40	100	*yhhY*	N->D
3770539	C	T	36	100	*yibH*	V->M
3241106	A	G	34	100	*sstT*	N->S
3737783	C	T	33	100	*malS*	T->I
3167148	G	A	32	100	*ygiS*	H->Y
534742	G	A	30	100	*gcl*	G->D
4259570	G	A	29	100	*zur*	A->V
3134479	C	T	29	100	*yghT*	Q->stop
3725176	T	G	28	100	*glyQ*	E->A
3486502	C	T	26	100	*crp*	T->S
2867455	G	A	26	100	*rpoS*	Q->stop
309381	C	T	25	100	*ecpB*	D->N
4473900	C	T	24	100	*bdcA*	G->R
3599487	C	T	23	100	*livJ*	A->T
3279276	C	G	23	100	*kbaZ*	F->L
4081749	G	A	22	100	*fdoI*	P->S
2587112	C	T	21	100	*narQ*	A->V
4549043	T	G	20	100	*fimH*	L->R
3411689	G	T	20	100	*yhdJ*	E->stop
3329743	G	T	20	100	*dacB*	D->Y
3296481	G	A	20	100	*yraP*	V->M
4325644	C	T	18	100	*yjdN*	S->N
3302338	C	A	18	100	*yhbU*	A->E
2556984	G	A	17	100	*eutC*	T->I
81102	G	A	17	100	*glpA*	S->L
2404402	G	A	16	100	*nuoB*	S->L
2247134	A	C	16	100	*lysP*	Y->D
1667460	G	A	16	100	*mlc*	Q->stop
3388041	T	G	15	100	*aaeB*	T->P
2744217	C	T	15	100	*rplS*	G->D
410666	C	T	15	100	*mak*	P->S
3602471	C	T	14	100	*ftsE*	D->N
2942306	G	A	14	100	*gcvA*	Q->stop
2040433	C	A	14	100	*yedY*	A->D
582597	C	T	14	100	*ybcY*	S->N
4133270	C	T	13	100	*metF*	Q->stop
3087508	C	T	13	100	*metK*	P->L
1894839	T	C	13	100	*pabB*	L->P
1077657	C	T	13	100	*putA*	R->H
700038	G	A	13	100	*umpH*	H->Y
4325000	C	T	12	100	*phnC*	G->S
2531659	C	T	12	100	*cysZ*	P->L
495622	C	T	11	100	*htpG*	T->I
85203	C	T	11	100	*leuO*	P->L
2028651	G	A	10	100	*yedI*	A->V
1643679	A	T	10	100	*ydfU*	L->Q
1301992	A	T	9	100	*oppA*	T->Y
1147792	T	A	9	100	*plsX*	S->T

156068	A	G	9	100	*yadV*	V->A
71170	T	C	9	100	*araC*	S->P
1301979	A	C	8	100	*oppA*	E->D
1797083	C	T	7	100	*pheT*	V->M
3465502	G	A	6	100	*gspM*	E->K
2017589	A	C	6	100	*fliI*	I->L
1306736	T	G	6	100	*oppF*	S->A
1169836	A	G	6	100	*ycfQ*	L->P
731392	A	G	6	100	*rhsC*	T->A
3474424	T	G	5	100	*rpsL*	K->N
2233609	C	T	4	100	*yeiS*	Q->stop
1105847	G	A	4	100	*ymdB*	G->S
456711	C	G	4	100	*clpP*	P->R
1652331	T	C	2	100	*intQ*	F->L
367708	A	C	129	88.36	*mhpR*	I->S
526151	C	T	14	82.35	*rhsD*	P->L
367691	T	C	49	71.01	*mhpR*	K->E
1405758	G	A	9	100		
4035426	C	T	8	57.14		
1996093	G	A	7	100		

Gene name	Function	Change in RP437 (location in MG1655)	Change in W3110 (location in MG1655)
crp	cAMP activated global transcription factor	T->S (3486502)	K->T (3486206)
rpoS	RNA polymerase, sigma S (sigma 38) factor	Q->stop (2867455)	
intQ	pseudo	F->L (1652331)	

Supplementary – An estimate for the oxygen gradient across the gel layer

In the following, we estimate the expected oxygen gradient across the thin gel layer under simplifying assumptions. We do not consider here the accumulation of bacteria at the gel/liquid interface, which would tend to enhance the gradient. In addition, we assume steady state with homogeneous distribution of cells in the chamber. However, the pre-equilibrated bacterial suspension is not static but, in fact, flowing through the chamber such that the content of the chamber is being replaced every ~ 10 min. This flow also limits the oxygen concentration in the chamber and thus tends to enhance the gradient.

Fig. 1 presents the model considered here. Oxygen enters on the left and maintains a certain concentration C_0 of dissolved oxygen at this interface. We assume that there are no cells in the gel region (length l) and thus in steady state the oxygen concentration is decreasing linearly with the distance x and reaching a value defined as C_1 at the gel/bacterial-suspension interface.

$$-l < x < 0 \qquad D\frac{\partial^2 C(x)}{\partial x^2} = 0 \qquad \Rightarrow \quad C(x) = C_0 - \frac{C_0 - C_1}{l} \cdot (x + l)$$

In the region $x > 0$, oxygen is being consumed by the cells. Assuming a linear degradation term, the oxygen concentration would decay exponentially, with a characteristic length L.

$$x > 0 \qquad D\frac{\partial^2 C(x)}{\partial x^2} - k \cdot C(x) = 0 \qquad \Rightarrow \quad C(x) = C_1 \cdot e^{-x/L} \qquad with \quad L = \sqrt{\frac{D}{k}}$$

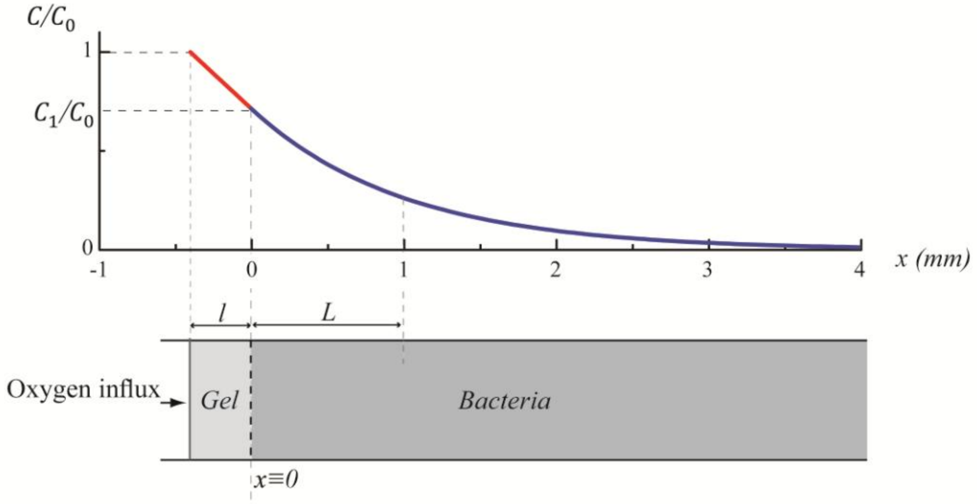

Fig. 1. The expected oxygen profile for $l = 0.4\ mm$ and $= 1\ mm$.

In steady state, C_1 is self-tuned to maintain the balance between the incoming oxygen flux at $x = 0$ and its overall consumption in the region $x > 0$. (This condition is equivalent to demanding flux continuity.)

$$J_{x=0} = \int_0^{\infty} k \cdot C(x)dx \qquad \Rightarrow \qquad D \cdot \frac{C_0 - C_1}{l} = \int_0^{\infty} k \cdot C_1 \cdot e^{-\frac{x}{L}} dx$$

leading to **$Q1$.** $$C_1 = C_0 \cdot \frac{1}{1 + {}^{l}/_{L}}$$

To estimate the decay length L, we introduced cell suspensions of either the MG1655 or MG1655(*aer*) strain into a capillary tube and measured the distance between the air/liquid interface and the place where the bacterial swimming comes to a halt. The transition from swimming to non-swimming cells appeared sharp, marking the position where the oxygen level is close to zero (see ref. 1). This distance was consistently 1.5-2 mm (up to 30 min after application), consistent with a decay length of approximately 1 mm.

L can also be evaluated from the bacterial consumption rate Q and the bacterial cell density ρ, which is continually flowing through the chamber. Note that the time scale $1/k$~5 min is similar to the chamber exchange rate (10 min.) due to the flow.

$$-\frac{\partial C}{\partial t} = k \cdot C = Q \cdot \rho \qquad \Rightarrow \qquad k = \frac{Q \cdot \rho}{C} \qquad \Rightarrow \quad L = \sqrt{\frac{D}{k}} = \sqrt{\frac{D \cdot C}{Q \cdot \rho}} \sim 0.6\, mm$$

Diffusion constant[2]	$D \sim 2 \cdot 10^{-5} \ \frac{cm^2}{s}$
Oxygen consumption rate[3]	$Q \sim 2 \cdot 10^{-18} \ \frac{Mole}{cell \cdot s}$ (20 *mmol/h/gdw* & 300 *fg/cell*)
Oxygen concentration	$C \sim 200 \cdot 10^{-6}\ Molar$;
Bacterial cell density (OD_{600}=1)	$\rho \sim 5 \cdot 10^{11} \ \frac{cells}{Liter}$

Thus, substituting $L{\sim}1mm$ and $l{\sim}0.4mm$ in $Q1$, we get $C_1 \sim 0.7 \cdot C_0$, suggesting that the oxygen concentration is expected to vary across the gel layer by more than 30%.

1 Douarche, C., Buguin, A., Salman, H. & Libchaber, A. *E. Coli* and Oxygen: A Motility Transition. *Phys. Rev. Lett.* **102**, 198101 (2009).

2 Fennema, O. & Kester, J. Resistance of lipid films to transmission of water vapor and oxygen. *Adv. Exp. Med. Biol.* **302**, 703-719. (1991).

3 Andersen, K. B. & von Meyenburg, K. Are growth rates of *Escherichia coli* in batch cultures limited by respiration? *J. Bacteriol.* **144**, 114-123 (1980).
